# Genome analysis of *Daldinia eschscholtzii* strains UM 1400 and UM 1020, wood-decaying fungi isolated from human hosts

**DOI:** 10.1186/s12864-015-2200-2

**Published:** 2015-11-18

**Authors:** Chai Ling Chan, Su Mei Yew, Yun Fong Ngeow, Shiang Ling Na, Kok Wei Lee, Chee-Choong Hoh, Wai-Yan Yee, Kee Peng Ng

**Affiliations:** Department of Medical Microbiology, Faculty of Medicine, University of Malaya, 50603 Kuala Lumpur, Malaysia; Department of Pre-Clinical Sciences, Faculty of Medicine and Health Sciences, University Tunku Abdul Rahman, Bandar Sungai Long, 43000 Kajang, Selangor Darul Ehsan Malaysia; Codon Genomics S/B, No 26, Jalan Dutamas 7, Taman Dutamas, Balakong, Seri Kembangan, 43200 Selangor Darul Ehsan, Malaysia

**Keywords:** Genome sequencing, *Daldinia eschscholtzii*, Wood-inhabiting endophyte, Wood-decaying fungi

## Abstract

**Background:**

*Daldinia eschscholtzii* is a wood-inhabiting fungus that causes wood decay under certain conditions. It has a broad host range and produces a large repertoire of potentially bioactive compounds. However, there is no extensive genome analysis on this fungal species.

**Results:**

Two fungal isolates (UM 1400 and UM 1020) from human specimens were identified as *Daldinia eschscholtzii* by morphological features and ITS-based phylogenetic analysis. Both genomes were similar in size with 10,822 predicted genes in UM 1400 (35.8 Mb) and 11,120 predicted genes in UM 1020 (35.5 Mb). A total of 751 gene families were shared among both UM isolates, including gene families associated with fungus-host interactions. In the CAZyme comparative analysis, both genomes were found to contain arrays of CAZyme related to plant cell wall degradation. Genes encoding secreted peptidases were found in the genomes, which encode for the peptidases involved in the degradation of structural proteins in plant cell wall. In addition, arrays of secondary metabolite backbone genes were identified in both genomes, indicating of their potential to produce bioactive secondary metabolites. Both genomes also contained an abundance of gene encoding signaling components, with three proposed MAPK cascades involved in cell wall integrity, osmoregulation, and mating/filamentation. Besides genomic evidence for degrading capability, both isolates also harbored an array of genes encoding stress response proteins that are potentially significant for adaptation to living in the hostile environments.

**Conclusions:**

Our genomic studies provide further information for the biological understanding of the *D. eschscholtzii* and suggest that these wood-decaying fungi are also equipped for adaptation to adverse environments in the human host.

**Electronic supplementary material:**

The online version of this article (doi:10.1186/s12864-015-2200-2) contains supplementary material, which is available to authorized users.

## Background

*Daldinia* spp. belonging to the phylum Ascomycota and class Sordariomycetes, are known as endophytes or latent pathogens which inhabit woody host plants and remain in a dormant phase until the decay of wood or formation of perithecial stromata [1]. The ascospores and conidia of these fungi are spread to neighboring trees via wind movements or fungivorous insects [2]. *Daldinia eschscholtzii* has been isolated from dead trees [3], marine alga [4], insect [5] and recently also from human specimens [6, 7], displaying the great adaptation ability of this organism in a diverse host range. To date, there is no extensive analysis of the *D. eschscholtzii* genome, although this fungal species has been shown to produce potential bioactive compounds.

Secondary metabolites produced by *D. eschscholtzii* have potential medical and industrial applications. Zhang et al. [5, 8] reported the isolation of immunosuppressive compounds, including dalesconol A, B and C, daeschol A, 2, 16-dihydroxyl-benzo[*j*]fluoranthene and dalmanol A, from mantis-associated *D. eschscholtzii*. Helicascolide C, a new lactone with fungistatic activity against *Cladosporium cucumerinum* was isolated together with helicascolide A from an Indonesian marine algicolous-associated *D. eschscholtzii* [4]. *Daldinia* spp. have been reported to produce volatile organic compounds (VOCs) [9–12] which can be developed in industrial applications for biofuel, biocontrol, and mycofumigation.

*D. eschscholtzii* has previously shown a typical feature of wood-decaying fungi, which is the production of enzymes for the degradation of lignocellulosic biomass, such as endoglucanase and β-glucosidase [3, 13]. As previously described, *Daldinia* spp. are known as type II soft-rot fungi that cause erosive degradation of lignocelluloses [14]; one sp. *D. concentrica* has been shown to be able to degrade the recalcitrant non-phenolic structures of lignin [15]. This indicates that *Daldinia* spp. may have the ability to convert lignocellulosic biomass into different value-added products including biofuel, chemicals, and cheap carbon sources for fermentation, improved animal feeds, and human nutrients.

In this study, we present the genome of *D. eschscholtzii* UM 1400, an isolate obtained from human skin scraping, that enabled us to perform detailed analysis with the previously published genome of *D. eschscholtzii* UM 1020 [6] for shared and common biological features. The genetic information of *D. eschscholtzii* UM 1400, combined with that of *D. eschscholtzii* UM 1020, will provide the knowledge for a deeper understanding of the biological nature of *D. eschscholtzii*.

## Results and discussion

### Morphological and molecular identification

**Fig. 1:**
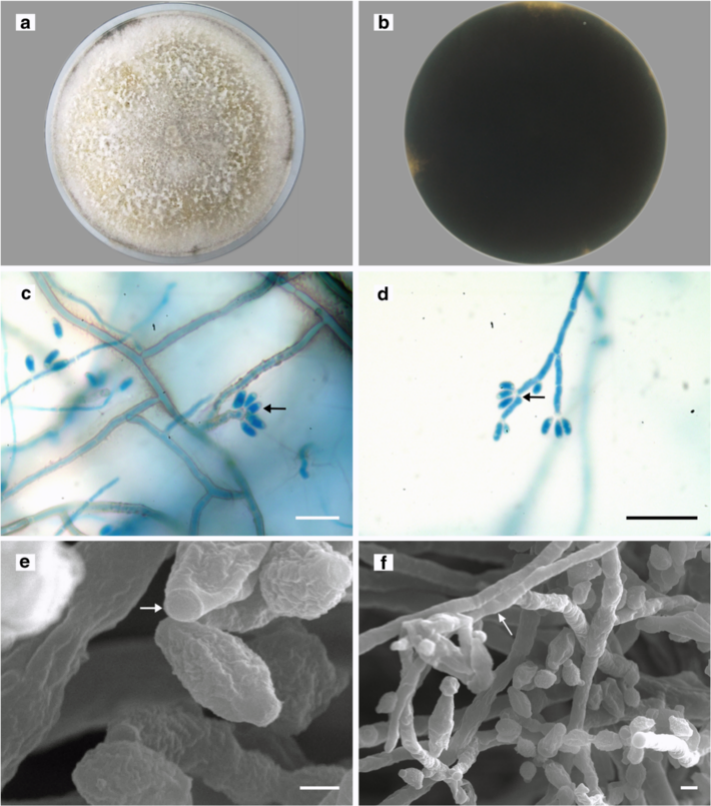
Morphological observations of the representative UM isolate. **a** Top view of culture on SDA plate for 6 days at 30 °C. **b** Black color from the reverse view of culture on SDA plate, indicating the presence of melanized hyphae. **c** Microscopic observation; the arrow shows the conidia produced in sympodial sequence. Bar scale is 20 μm. **d** Microscopic observation; the arrow shows the conidiophore branching from the conidiogenous area. Bar scale is 20 μm. **e** Scanning electron micrograph; the arrow shows the conidium with flattened base. Bar scale is 1 μm. **f** Scanning electron micrograph; the arrow shows the rough surface of conidiophore. Bar scale is 2 μm

UM 1400 and UM 1020 isolates (UM isolates) were grown on Sabouraud dextrose agar (SDA) incubated at 30 °C for 6 days. Both cultures initially appeared as whitish, azonate and felty colonies with diffuse margins, and later became smoky gray with slight olivaceous tones (Fig. 1a). The reverse side of culture plate appeared dark in color, indicating the growth of melanized hyphae (Fig. 1b). Microscopic observation under the light microscope showed septate conidiophores mononematously or dichotomously branched with conidiogenous cells arising from the terminus. From the apical end of conidiogenous cells, conidia were produced holoblastically in sympodial sequence (Fig. 1c and d). The conidiophore was also observed to be branched from the conidiogenous area (Fig. 1d). The surface topology of conidiophores and conidia was examined under the scanning electron microscope (SEM). Coarsely rough conidiophores, as well as ellipsoid-shaped rough conidia with a flattened base (Fig. 1e and f), were seen. These anamorphic features are similar to those of the *Daldinia* spp. previously described by Ju et al. [16].

**Fig. 2:**
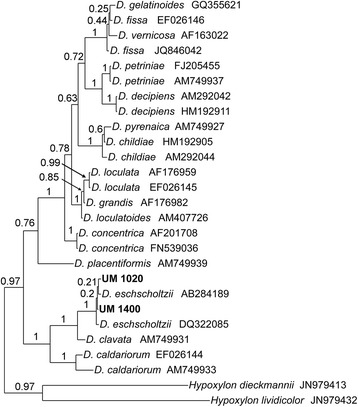
ITS rDNA-based phylogenetic relationship of both UM 1400 and UM 1020 with other *Daldinia* species. The ITS sequences of all *Daldinia* species were obtained from the GenBank database and the phylogenetic analysis was performed using MrBayes version 3.2.1. UM 1400 and UM 1020 are indicated in bold font

The identities of both UM isolates were indicated by their ITS rDNA sequence similarity to other *D. eschscholtzii* strains, as well as ITS-based phylogenetic analysis that showed both UM isolates clustering with *D. eschscholtzii* reference strains (Fig. 2). The complete ITS sequences of *D. eschscholtzii* UM 1400 and UM 1020 were deposited in the GenBank database under the accession numbers [GenBank: JX966561 and JX966563], respectively.

### Genome sequencing and assembly

The UM 1020 genome was sequenced with Illumina Genome Analyzer IIx as previously reported [6]. It was sequenced and assembled using a single 350 bp insert size genomic DNA library that generated 123-fold coverage of Illumina reads with a total genome size of 35.5 Mb (Table 1). In this study, the UM 1400 genome was sequenced to 106-fold depth on Illumina HiSeq 2000 and assembled using a combination of two different insert size (500 bp and 5 kb) genomic DNA libraries. In this genome assembly, we were able to gap close many small contigs and link them together into bigger scaffolds, especially in the repetitive sequence regions, by utilizing sequencing reads from the 5 kb insert size reads library. This assembly resulted in 104 scaffolds (35.8 Mb), a significant reduction from the 598 scaffolds in the UM 1020 assembly, but with 1295 more contigs. The UM 1400 and UM 1020 genomes (UM genomes) showed similar GC content (46.51 and 46.80 %) and the number of predicted coding genes (10,822 and 11,120).

**Table 1:** Summary of the assembly and annotation features of *Daldinia eschscholtzii* UM 1400 and UM 1020

Features	*Daldinia eschscholtzii*	
	UM 1400	UM 1020
Reads from small-insert library (Gb)	2.32	4.38
Reads from large-insert library (Gb)	1.48	-
Total Reads (Gb)	3.80	4.38
Assembly size (Mb)	35.8	35.5
Number of contigs (≥200 bp)	1939	640
Contigs size (N50) (bp)	33,562	112,742
GC content of contigs (%)	46.80	46.81
Number of scaffolds (≥1000 bp)	104	598
Scaffolds size (N50) (bp)	701,334	114,605
GC content of scaffolds (%)	46.51	46.80
Number of predicted genes (≥99 bp)	10,822	11,120
Average gene length (bp)	1483	1616
Average number of exons per gene	2.87	2.82
CEGMA completeness score calculated from the complete gene set (%)	94.76	96.37
CEGMA completeness score calculated from both complete and partial gene sets (%)	96.77	97.18
rRNA	29	28
tRNA	168	156
Repetitive sequence (%)	1.02	1.42
Functional annotation^a^	3357	1974
KEGG	975	998
GO	6471	6223
KOG	6168	6195
Pfam	7690	7725

^a^Annotation from SwissProt and NR (for genes without SwissProt hits) with no keywords of predicted, unknown, unnamed and hypothetical

The completeness of genome assembly was assessed using the CEGMA (Core Eukaryotic Genes Mapping Approach) software that evaluates the presence and completeness of a widely conserved set of 248 core eukaryotic genes [17, 18]. The standard CEGMA pipeline identified 235 out of the 248 core eukaryotic genes (94.76 %) in the UM 1400 genome assembly as complete, with an additional five core eukaryotic genes detected as partial (2.02 %). Similarly, out of the 248 core eukaryotic genes, a total of 239 (96.37 %) complete and two (0.81 %) partial core eukaryotic genes were detected when assessing the genome assembly of UM 1020 isolate. These results from CEGMA indicate that both assembled genomes cover most of the eukaryote’s gene space with many of genes complete and not fragmented onto multiple contigs.

The percentage of the repetitive sequences in both genomes (1.02 % in UM 1400 and 1.42 % in UM 1020) was lower than that reported for other Sordariomycetes genomes, for instance, 9.77 % in *Magnaporthe grisea* [19] and 10 % in *Neurospora crassa* [20]. Of the repetitive sequences, transposable elements comprised 0.12 and 0.14 % in the UM 1400 and UM 1020 genomes, respectively. The transposable elements were classified into eight (UM 1400) and 12 (UM 1020) families with the subclass of Ty1_Copia most abundant in UM 1400 and the subclass of ISC1316 most abundant in UM 1020 (Additional file 1: Table S1). These data suggest that the *D. eschscholtzii* genomes are poor in repetitive sequences. However, it has been reported that repeat contents in Illumina-sequenced genomes are likely to be underestimated owing to a difficulty with the assembly of short repetitive reads into long repeat regions [21]. Hence, the low repeat content of UM genomes is probably due to the Illumina technology that generates short reads that are prone to errors in the estimation of repetitive sequences, especially when the repetitive sequences are longer than the length of the sequencing reads [22–25].

**Fig. 3:**
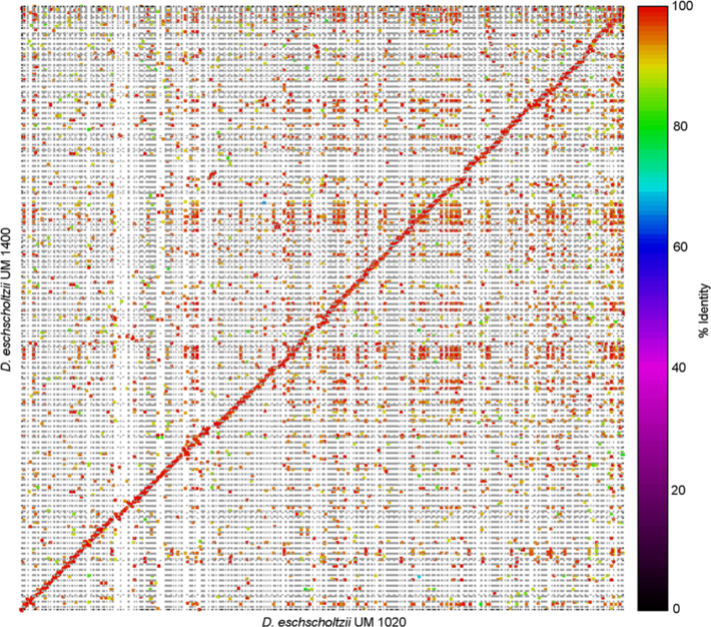
Dot-plot representing whole genome comparison between UM 1400 and UM 1020 isolates. The comparison was performed using MUMmer 3.23 (NUCmer). Dots closest to the diagonal line represent co-linearity between the two genomes. The dots are color-coded as indicative of percent sequence identity. The red dots or diagonal line depicts the high sequence identity between both genomes

The whole genome comparison between both UM isolates was performed using the NUCmer pipeline of the MUMmer software and visualized in dot-plot generated by mummerplot [26]. The generated synteny dot-plot showed the co-linearity between the two genomes and high levels of sequence homology to each other with more than 95 % sequence identity (Fig. 3). This reveals a macrosyntenic conservation pattern of gene content within both *D. eschscholtzii* genomes.

The genome sequence of *D. eschscholtzii* UM 1400 has been deposited in the European Nucleotide Archive (ENA) under the accession numbers [ENA: CCED01000001-CCED01001944 and LK023387-LK023490]. The version described in this paper is the first version of this genome sequence.

### Phylogenomic analysis

**Fig. 4:**
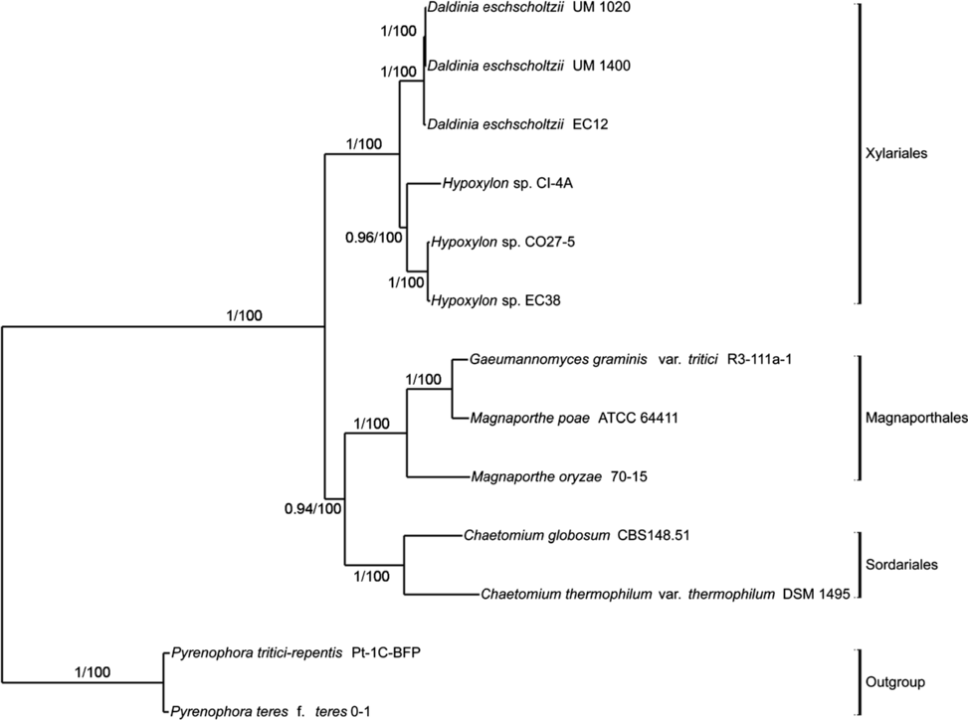
Phylogenomic position of both UM 1400 and UM 1020 within class Sordariomycetes fungi. The phylogenomic tree was constructed based on orthologous proteins from 11 Sordariomycetes genomes and two Dothideomycetes genomes as outgroups using both Maximum Likelihood and Bayesian methods

Nine Sordariomycetes genomes and two outgroups from Dothideomycetes (Additional file 1: Table S2) were used for phylogenomic analysis with our UM isolates of *D. eschscholtzii*. A total of 151,536 proteins were clustered into 18,771 orthologous families with 3322 single-copy orthologs identified. Concatenated alignments of 332 (~10 %) single-copy orthologs were used to generate Maximum Likelihood and Bayesian trees. Congruence was achieved by both trees with the Sordariomycetes genomes grouped into three orders of Xylariales, Magnaporthales, and Sordariales (Fig. 4). UM 1400 and UM 1020 clustered with the Xylariales and formed a monophyletic group with *D. eschscholtzii* EC12, which is an endophyte associated with the rainforest tree *Myroxylon balsamum* found in the upper Napo region of the Ecuadorian Amazon [9].

### Gene families

**Fig. 5:**
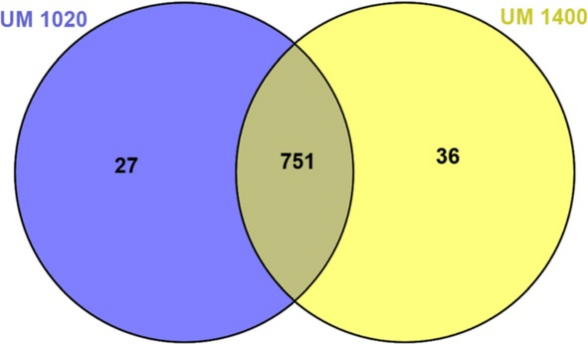
Venn diagram showing the number of shared gene families in both UM isolates. Summary of the number of shared gene families in UM 1400 (yellow) and UM 1020 (blue) among 15,691 gene families generated from all the Sordariomycetes genomes. The numbers in the circles represent number of different sets of gene families that are shared by both isolates and unique to each isolate

All predicted protein coding genes were analyzed using the OrthoMCL program to identify core gene families in the Sordariomycetes fungi (Additional file 1: Table S2) and our UM isolates of *D. eschscholtzii*. Of the 15,691 gene families identified, 751 (4.78 %) were shared by both UM 1400 and UM 1020 (Fig. 5; Additional file 1: Table S3). Among these 751 shared gene families were 182 clusters with known functions, 16 with unknown functions, and 553 without annotations in the database (Additional file 1: Table S4). The most abundant gene families shared by the two UM isolates were those encoding cytochrome P450 (13 clusters), major facilitator superfamily (nine clusters) and the heterokaryon incompatibility protein (eight clusters). These protein families are likely to play an important role in fungus-host interactions, as cytochrome P450 proteins detoxify host defense compounds, major facilitator superfamily transporters export secondary metabolites and host-derived antimicrobial compounds, and the heterokaryon incompatibility proteins control vegetative reproduction to produce viable heterokaryons necessary for the adaptation to environment and to host defense mechanisms.

The other shared gene families associated with fungus-host interactions included genes encoding the CFEM domain-containing protein (family SORD10851), FAS1 domain-containing protein (family SORD11403), and polysaccharide lyase family 4 protein (family SORD11288). The CFEM and FAS1 domains are present in fungal membrane proteins that could function as cell surface receptors or adhesion molecules in interactions with the host [27, 28]. The conservation of these genes suggests that both UM isolates may encode specific cell surface proteins with important roles in the interaction with its specific host. The polysaccharide lyase family 4 protein (rhamnogalacturonan lyase) cleaves the backbone of rhamnogalacturonan-I, which is a major component of plant cell wall polysaccharide pectin. A previous study showed that no endophytes tested have the ability to degrade pectin, and suggested that an endophyte is likely to be a latent pathogen if it can degrade pectic substances [29]. Hence, the presence of gene encoding rhamnogalacturonan lyase indicates the ability of both isolates to produce pectic enzyme for pectin degradation. In line with this, it is implied that both UM isolates are likely to be latent pathogens, a lifestyle of *D. eschscholtzii* as described previously by another research group [1].

Both UM isolates shared gene families associated with stress response, for instance, genes encoding acid trehalase (family SORD14461) and ClpB protein (family SORD11060). The acid trehalases are involved in the assimilation of extracellular trehalose as a carbon source under nutrient limitation, as previously revealed in acid trehalase-deficient mutants of *Saccharomyces cerevisiae* and *Aspergillus nidulans* [30, 31]. The ClpB protein is an ATP-dependent molecular chaperone that plays an essential role in disaggregation and reactivation of the aggregated proteins in response to heat stress [32]. These stress responses are necessary for fungal survival and adaptation in harsh environmental conditions.

### Plant cell wall degrading enzymes

Plant cell wall degradation contributes to the nutrient availability for fungal growth, and fungal penetration into host cells [33]. Generally, the fungal enzymes involved in plant polysaccharide degradation are assigned to the classes of glycoside hydrolase (GH), carbohydrate esterase (CE) and polysaccharide lyase (PL) in the CAZyme database. Both UM isolates were found to contain carbohydrate-active enzymes (CAZymes) specifically for plant polysaccharide degradation, with a total of 283 and 292 putative functional domains identified in the UM 1400 and UM 1020 genomes respectively (Table 2; Additional file 1: Table S5). These numbers of CAZyme domains were not far-off from those reported previously [33] in the facultative pathogen *Aspergillus fumigatus* (299), biotrophic fungus *Cladosporium fulvum* (315), hemibiotrophic fungus *Fusarium graminearum* (321), and hemibiotrophic fungus *Magnaporthe oryzae* (292) (Table 2; Additional file 1: Table S5).

**Table 2:** Comparison of total number of CAZymes with enzymatic activity for plant polysaccharide degradation

Fungal species	Lifestyle	CAZymes			
		C	HC	P	Total
CT	Saprophyte	23	60	42	125
NC		29	76	62	167
AF	Facultative Pathogen	46	127	126	299
CG		40	121	92	253
TM	Symbiont	16	34	27	77
ClG		15	54	41	110
BG	Biotroph	4	15	13	32
CF		45	133	137	315
FG	Hemibiotroph	47	136	138	321
MO		46	140	106	292
MP	Necrotroph	41	113	89	243
GG		49	128	95	272
UM 1400	Endophyte/wood decaying fungi	43	126	114	283
UM 1020		43	131	118	292

The number of CAZymes for plant polysaccharide degradation identified in UM 1400 and UM 1020 genomes was compared with those in the selected ascomycetous fungi of different lifestyles (summarized from Additional file 1: Table S5). Note that the CAZyme referred here indicates functional modules or domains but not genes. Data obtained from Zhao et al. [32]. Enzyme abbreviations: C cellulase, HC hemicellulase, P pectin. Fungal species abbreviations: *CT Chaetomium thermophilum*, *NC Neurospora crassa*, *AF Aspergillus fumigatus*, *CG Chaetomium globosum*, *TM Tuber melanosporum*, *ClG Cladonia grayi*, *BG Blumeria graminis*, *CF Cladosporium fulvum*, *FG Fusarium graminearum*, *MO Magnaporthe oryzae*, *MP Magnaporthe poae*, GG *Gaeumannomyces graminis*

In our UM genomes, we identified functional domains of a) three classes of cellulase for the complete degradation of cellulose (β-1,4-endoglucanase of CAZyme families GH5, GH7 and GH45, cellobiohydrolase of CAZyme families GH6 and GH7, and β-glucosidase of CAZyme families GH1 and GH3); b) hemicellulase for the degradation of xylan (β-1,4-endoxylanase of CAZyme families GH10 and GH11, and β-1,4-xylosidase of CAZyme families GH3 and GH43), xyloglucan (xyloglucanase of CAZyme families GH12 and GH74) and mannan (β-1,4-endomannanase of CAZyme family GH5 and β-mannosidase of CAZyme family GH2); c) pectinases (endo-polygalacturonase of CAZyme family GH28, exo-polygalacturonase of CAZyme family GH28, α-rhamnosidase of CAZyme family GH78, unsaturated glucuronyl hydrolase of CAZyme family GH88, pectate lyase of CAZyme family PL1, and rhamnogalacturonan lyase of CAZyme family PL4) and d) lignin-degrading enzymes of which, CAZyme families AA3 (glucose/methanol/choline oxidoreductases) and AA7 (glucooligosaccharide oxidase) appeared to be present in larger numbers than in other wood-decaying fungi like the white rot fungus *Phanerochaete chrysosporium* and brown rot fungus *Postia placenta* [34] (Table 3). As previously described by Levasseur et al*.* [34], the AA3 family is prevalent in some soft rot fungi from the Ascomycota group. The family AA3 enzymes are known to provide hydrogen peroxide required by the family AA2 enzymes (class II peroxidases) for catalytic activity whereas family AA7 enzymes are known to be involved in the biotransformation or detoxification of lignocellulosic biomass [34]. Generally, the families AA1 enzymes (multicopper oxidase) and AA2 enzymes (class II peroxidase) are the main oxidative enzymes that degrade phenolic and non-phenolic structures of lignin. The small number of these enzymes identified in the UM genomes indicates low oxidation activity for the degradation of lignin structure.

**Table 3:** Comparison of total number of CAZymes with ligninolytic auxiliary activities

Families	Known activities	PC	PP	UM 1400	UM 1020
AA1	Multicopper oxidase	1	3	3	3
AA2	Class II peroxidase	16	0	5	5
AA3	GMC oxidoreductase	8	7	29	29
AA4	Vanillyl alcohol oxidase	0	0	6	6
AA5	Radical-copper oxidase	7	2	1	1
AA6	1,4-benzoquinone reductase	4	1	2	2
AA7	Glucooligosaccharide oxidase	0	0	41	41
AA8	Iron reductase domain	2	0	4	4

The number of CAZymes with ligninolytic auxiliary activities identified in UM 1400 and UM 1020 genomes was compared with those in the white rot fungus *Phanerochaete chrysosporium* and brown rot fungus *Postia placenta* (data from Levasseur *et al.* [33]). Note that the CAZyme referred here indicates functional modules or domains but not genes. Fungal species abbreviations: *PC Phanerochaete chrysosporium*; *PP Postia placenta*

The presence of CAZymes with enzymatic activities for plant cell wall degradation implies that both human host-isolated *D. eschscholtzii* have once lived in the environment as wood-decaying fungi with degrading ability on plant biomass. These CAZymes are suggested to be required to degrade the wood cell consisting of the primary cell wall, secondary cell wall, and middle lamella, with each cell component containing different ratios of cellulose, hemicellulose, pectin and lignin. In addition, we identified six functional domains of cutinase (CAZyme family CE5) in the UM 1400 genome and five in the UM 1020 genome. Cutinases are critical for the initial fungal penetration through the cuticular barrier attached to the epidermal cell walls in aerial parts of plants, such as leaves, flowers, fruits and young stems [35]. This indicates that both UM isolates have the potential ability to penetrate through not only lignified woody cell walls but also plant cuticle and epidermal cell walls as well.

### Secondary metabolites

The wood-inhabiting endophyte *D. eschscholtzii* has been reported to produce arrays of secondary metabolites that have potential applications in medical and biofuel industries, such as immunosuppressive polyketides and volatile organic compounds [5, 8–10]. In the UM 1400 and UM 1020 genomes, we identified 47 and 45 secondary metabolite backbone genes respectively, including those encoding lovastatin nonaketide synthase, conidial pigment polyketide synthase Alb1, dimethylallyl tryptophan synthase (DMATS) and citrinin polyketide synthase (Additional file 1: Table S6).

Lovastatin nonaketide synthase is involved in the biosynthesis of lovastatin, a cholesterol-lowering drug [36]. The presence of this encoding gene suggests that both UM isolates may produce essential enzyme needed to manufacture the potent drug lovastatin for lowering blood cholesterol. Another polyketide synthase, Alb1, is responsible for the heptaketide naphtopyrene YWA1 synthesis in conidial pigmentation. Tsai et al. [37] reported that *Aspergillus fumigatus* produces Alb1 protein to synthesize the conidial pigment via the pentaketide pathway. This indicates that heptaketide synthase may be involved in the initiation of pentaketide melanin biosynthesis in *D. eschscholtzii*. Melanin protects fungal spores and mycelium against environmental stresses, including desiccation, oxidizing agents and ultraviolet (UV) light. Thus, melanin production may be a protective trait that allows *D. eschscholtzii* to survive in harsh conditions such as drought that triggers desiccation and osmotic stress.

Dimethylallyl tryptophan synthase (DMATS) and citrinin polyketide synthase are involved in the synthesis of ergot alkaloids and antibiotic citrinin, respectively. As previously reported, ergot alkaloids were shown to be poisonous to herbivores [38], while citrinin had antimicrobial activity against pathogens [39]. These bioactive compounds may play a similar role in both UM isolates to confer beneficial protection to its host plant from the attacks of herbivores and pathogens subjected to further confirmation. A previous study reported that an endophytic *Daldinia eschscholtzii* EC12 produces volatile organic compounds that are active against a broad range of plant pathogens [9, 10].

### Secreted peptidases

Secreted peptidases facilitate fungal penetration and colonization of the host plant by degrading plant cell wall structural proteins and plant defense-related proteins [40, 41]. Examples are subtilisin-like peptidases (MEROPS subfamily S08A) and metallopeptidases (MEROPS families M35 and M36).

Subtilisin-like peptidases are serine peptidases that have been found to be associated with colonization of the host by endophytes [42] and plant pathogenic fungi [40]. We identified 15 genes (eight in the UM 1400 genome and seven in the UM 1020 genome) encoding subtilisin-like peptidases (Additional file 1: Table S7). The metallopeptidases are known to cleave the glycoproteins of extracellular matrix that have been implicated in host resistance mechanisms against pathogen invasion [43, 44]. For instance, the fungalysin of M36 family was shown to truncate non-structural host resistance proteins [41]. The presence of genes encoding penicillolysin of M35 family and fungalysin of M36 family in both genomes indicates the ability of UM isolates to inactivate proteinaceous components from the plant defense response.

### Pathogenicity-associated genes

A protein blast analysis against the pathogen-host interaction database (PHI-base) revealed 602 and 606 putative PHI genes (>50 % identity; >70 % subject coverage) in the genomes of UM 1400 and UM 1020 respectively. With the Eukaryotic Orthologous Group (KOG) functional classification, the putative PHI genes were distributed into 22 functional categories with a higher number assigned to the category of signal transduction mechanisms (Additional file 2: Figure S1).

### Signal transduction

The UM genomes contained arrays of putative genes encoding signaling components, and here, we discuss those involved in mitogen-activated protein kinase (MAPK) signaling pathways. The MAPK signaling pathways are commonly found in all eukaryotes and are known to be involved in cell growth, differentiation, and stress response. From the genomic analysis, three putative MAPK signaling pathways were proposed to be present in *D. eschscholtzii* UM 1400 and UM 1020 isolates, including the cell wall integrity pathway mediated by the Ssk2/Ssk22-Pbs2-Osm1 cascade, the osmoregulation pathway mediated by the Bck1-Mkk1/Mkk2-Mps1 cascade, and the mating/filamentation pathway mediated by the Mst11-Ste7-Gpmk1 cascade (Additional file 1: Table S8).

Numerous homologs of components of the osmoregulation pathway were identified in the UM genomes, including Sln1, Hik1, Sho1, Cdc42, Mst20 (Ste20 homolog), Mst50 (Ste50 homolog), Mst11 (Ste11 homolog), Pbs2, Osm1 (Hog1 homolog), and Ssk2/Ssk22. The *osm1* gene was shown to encode a functional homolog of MAPK Hog1 and to be required in response to osmotic stress [45]. As referring to the high-osmolarity glycerol (HOG) pathway in *Saccharomyces cerevisiae* [46], two upstream branches were predicted to activate the MAPK Pbs2-MAPK Osm1 module in both UM isolates. One branch consisted of MAPKK Ssk2/Ssk22 and a two-component histidine kinase phospho-relay system Sln1-Ypd1-Ssk1, with lacks homologs Ypd1 and Ssk1; another branch consisted of a putative membrane protein Sho1, Cdc42, Mst11, Mst20, and Mst50. Besides osmotic stress, the osmoregulation pathway is also required for adaptation to oxidative stress, thermal stress, cellular morphogenesis regulation, and cell wall functionality [47].

The UM genomes contained several homologs of components of the cell wall integrity pathway, including the GTP-binding protein Rho1, MAPKKK Bck1, MAPKK Mkk1/Mkk2, and MAPK Mps1 (Slt2 homolog). Generally, this MAPK pathway is essential for cell wall integrity and pathogenesis [46]. Some MAPK Slt2 homologs are also involved in other roles, such as conidium germination and polarized growth in *Aspergillus nidulans* [48], and response to various stresses, including oxidative and osmotic stresses, in *Candida albicans* [49].

The homolog of MAPK Gmpk1 of the mating/filamentation pathway was identified in both UM genomes. In *Fusarium graminearum*, Gmpk1 is required to regulate mating, conidial production, and pathogenicity as well as the early induction of extracellular endoglucanase, xylanolytic, proteolytic and lipolytic activities [50, 51]. Other identified homologous components of this pathway included a MAPKKK Mst11 (Ste11 homolog), a MAPKK Ste7, an adaptor protein Ste50, a PAK kinase Mst20 (Ste20 homolog), two small GTP-binding proteins Ras2 and Cdc42, a Gα subunit Gpa2. These components were previously reported to be involved in the activation of the mating/filamentation pathway in well-characterized *Saccharomyces cerevisiae* [52]. However, the homolog of G protein-coupled, seven-transmembrane receptor Gpr1 was not found in the UM genomes. One Ste12-like transcription factor, Cst1 homolog was identified in the UM genomes. As previously reported, this downstream transcription factor regulates genes involved in penetration and infectious growth in *Colletotrichum lagenarium* [53].

Although the MAPKKK-MAPKK-MAPK cascades are generally conserved in eukaryotes, the UM isolates seemed to lack significant homologs of upstream protein kinases, such as Ypd1 and Ssk1 in the osmoregulation pathway, and Gpr1 in the mating/filamentation pathway. This suggests that the upstream components in our UM isolates may be different from those in other well-characterized organisms, like *S. cerevisiae* [52], and may be novel receptor kinases for sensing environmental signals.

### Adaptation-associated stress response proteins

*Daldinia eschscholtzii* has been isolated from diverse environments [3–7] where it may be subjected to many extreme conditions. The transition from a moderate environment to a hostile environment causes drastic changes in various parameters, including osmotic changes, pH changes, thermal changes, nutrient deprivations, as well as oxidative and nitrosative stresses. In both UM genomes, we identified numerous stress-responsive genes as listed in Table S9 in Additional file 1.

*Daldinia* spp. appear to be adapted to survive during periods of drought in the natural environment, and even when their woody host plant has been fire-damaged [54]. These harsh conditions result in osmotic and thermal stresses to *Daldinia* spp. To maintain cellular turgor and prevent water loss, high concentrations of osmolytes, like glycerol, erythritol, mannitol, or trehalose are generated. The gene encoding osmotic stress-responsive proteins were identified in the UM genomes, including *os-4* orthologue, *os-1/nik-1* orthologue, and *pbsA* orthologue that are involved in osmolytes accumulation, *tpsA* and *orlA* that are involved in osmolyte trehalose biosynthesis, and *gfdB* that is involved in osmolyte glycerol biosynthesis. The increased production of osmolytes could induce the formation of vegetative structures conferring resistance to drought condition [55], likes the stromatic structures formed by *Daldinia* spp. for survival in drought [54]. Numerous genes encoding thermal stress-responsive proteins were found in the genomes, for instance, genes encoding heat shock proteins (*hsp70*, *hsp78*, *hsp104*, *hsf1*) which are induced to refold or degrade damaged proteins, to unfold aggregated proteins, and also to help in stabilizing proteins and membranes [47]. Unceasing wood decay will change the chemical composition and physical structure of wood which will, in turn, lead to nutrient deprivation stress. To tolerate this stressful condition, the alternate nutrient sources may be assimilated by expressing the genes associated with sources metabolism and nutrients uptake, such as *treB*, *mep1*, *mep2*, *prnB*, and *prnC*. The *treB* gene encodes a neutral trehalase that partially contributes to the energy requirements of spore germination under carbon limitation, as shown in the *tre* mutant of *Aspergillus nidulans* [56]. The *mep1* and *mep2* genes are the examples of genes encoding proteins involved in nitrogen assimilation, and are predominantly expressed at low concentrations of ammonium or on poor nitrogen sources. The ammonium permease encoded by the *mep2* gene has been shown to control nitrogen starvation-induced filamentous growth in *Candida albicans* via interaction with Ras1 [57]. In addition, fungi are able to utilize amino acids as sole nitrogen and/or carbon sources. In response to amino acid starvation, the transcription of the genes involved in amino acid biosynthesis are activated, such as *prnB* and *prnC* genes [58], both of which were found in the UM genomes.

Reactive oxygen species (ROS) and reactive nitrogen species (RNS) produced by hosts are harmful to fungi by causing damage to their proteins, lipid membranes, and deoxyribonucleic acid [59]. In order to survive in this harsh environment, fungi must have mechanisms to detoxify these reactive molecular species and repair the cellular damages triggered by the oxidative and nitrosative stresses. The UM isolates were found to contain genes encoding antioxidant enzymes (*sod1*, *sod2*, *sodA*, *cat1*, *tsa1*, *tsa3*, *grx5*, *gpxA*, *msrA*, *msrB*) and enzymes involved in the production of secondary metabolites with antioxidant function (*tpsA*, *orlA*) to handle ROS, as well as the gene encoding nitrosative stress-responsive proteins (*fhbA*) to cope with RNS. The plant pathogenic fungus *Botrytis cinerea* has been reported to produce these proteins to thrive against the oxidative and nitrosative environments generated by host plant cells [60, 61]. These enzymes have also been implicated in the defense of opportunistic fungal pathogens (*Candida albicans*, *Cryptococcus neoformans* and *Aspergillus fumigatus*) against the ROS and RNS produced by human phagocytes [62]. In the case of wood-decaying fungi, the extracellular hydrogen peroxide provided by oxidative enzymes is involved in the generation of highly reactive oxidants or hydroxyl radicals via the Fenton reaction with the presence of iron cofactor [63]. These radicals are involved in the degradation process. The high level of generated ROS is coped by the intracellular antioxidant enzymes to prevent fungal cell damage, as investigated in the previous study on the endogenous oxidative stress response of *Coriolus versicolor* [64]. Our CAZymes analysis identified oxidative enzymes in both UM genomes, with GMC oxidoreductases (family AA3) present in a high number (Table 3). This GMC oxidase (family AA3) has been thought to play an important role in peroxide production in the wood-decaying fungus *Gloeophyllum trabeum* [65]. Other enzymes, the copper radical oxidase (family AA5), FAD-linked oxidoreductase (family AA3) and glucose oxidase-like protein (family AA3) have been demonstrated to be potentially involved in extracellular peroxide production in *Postia placenta* [66].

The Fenton reaction requires iron as the cofactor of peroxidase enzymes for degradation activity. However, iron is sequestered by high-affinity iron binding proteins; thus, the iron acquisition system is required for wood-decaying fungi under iron starvation condition [67]. The UM genomes featured genes involved in iron acquisition, namely genes encoding iron permease (*Ftr1* orthologue) and mitochondrial ornithine carrier (*AmcA* orthologue). The Ftr1 protein is required for high-affinity iron uptake in the reductive iron uptake system [68], while the AmcA protein is involved in the supply of ornithine for siderophore biosynthesis [69]. The Ftr1 protein was shown to be up-regulated during the growth of *P. placenta* on cellulose medium [63].

Changes in pH can be encountered upon environmental transition. The gene encoding Pac1 ortholog of *Fusarium graminearum* was identified in the UM genomes. This gene has been previously reported to encode a pH regulator factor regulating the production of secondary metabolite in *F. graminearum* [70]. Overall, the UM isolates harbored many genes encoding stress response proteins that cope with triggered stresses under adverse conditions in their natural habitats. This feature could also serve to their advantage in surviving the adverse microenvironments of human niches.

## Conclusions

The genomic analysis of both UM isolates revealed a common set of putative domains or genes that improves our understanding of the biological nature of *D. eschscholtzii*. The environmental origin of these isolates is suggested by the identification of putative CAZyme arrays and genes encoding secreted peptidases related to plant cell wall degradation. As *D. eschscholtzii* has hitherto never been associated with human infections, our UM isolates might have been entering into humans via the exposure of open wounds to the decaying wood material containing this organism and have been surviving in the human without causing any disease. Both UM genomes displayed a wide range of adaptation-associated stress response genes that are required by fungi for adaptation to hostile conditions in their natural habitat. These genes most likely also confer a selective advantage for survival and adaptation in adverse microenvironments in the human host. Our genomic analysis also revealed other biological features, such as the identified genes encoding MAPK signaling pathway components that suggest three MAPK signaling cascades, and the identified secondary metabolite backbone genes that indicate the potential of the UM isolates to produce various bioactive secondary metabolites. The biological functions of predicted genes have to be validated by further studies using appropriate approaches such as insertional mutagenesis, serial analysis of gene expression, microarray analysis, proteomics, and metabolomics.

## Methods

### Ethical statement

As no patient information is disclosed, it was considered unnecessary to apply for ethical approval from the University Malaya Medical Centre (UMMC) Medical Ethics Committee (http://umresearch.um.edu.my/doc/File/UMREC/6_CODE%20OF%20RESEARCH%20ETHICS%20%20IN%20UNIVERSITY%20OF%20MALAYA.pdf).

### Fungal isolates

Both UM 1400 and UM 1020 isolates were recovered from a collection of fungi routinely cultured and archived in the Mycology diagnostic laboratory, UMMC. These isolates were grown on SDA plates at 30 °C and maintained on SDA slants at 4 °C until required for research use.

### Morphological identification

Fungal cultures on SDA were observed for cultural characteristics. Slide cultures mounted with lactophenol cotton blue stain were observed under the light microscope for anamorphic structures. For SEM examination, the cultures were fixed, dried, mounted on a specimen stub using electrically conductive double-sided adhesive tape, and sputter-coated with gold before observing under the XL-30 ESEM microscope (Philips, Netherlands) for the surface topography of conidia and conidiophores.

### Molecular identification

Fungal DNA was extracted using ZR Fungal/Bacterial DNA MiniPrepTM (Zymo Research, USA), according to the manufacturer’s protocol. The specific primer pair ITS1 and ITS4 was used to amplify the region of ITS1-5.8S-ITS2 rDNA, as previously described [71]. PCR products were visualized by gel electrophoresis and purified prior to Sanger sequencing. ITS sequences were searched against the NCBI nucleotide database to determine fungal identity. For phylogenetic tree analysis, the complete ITS1-5.8S-ITS2 sequence was collected from each *Daldinia* species available in the GenBank database. Multiple sequence alignments of all data-mined ITS sequences were generated using M-Coffee [72] which uses other packages to compute the alignments and uses T-Coffee to combine all of these alignments into one unique final alignment. Phylogenetic analysis was then performed using MrBayes version 3.2.1 [73]. Bayesian Markov Chain Monte Carlo (MCMC) analysis was performed by sampling across the entire general time reversible (GTR) model space. A total of 250,000 generations were run with a sampling frequency of 100, and diagnostics were calculated for every 1000 generations. A burn-in setting of 25 % was used to discard the first 625 trees. Convergence was assessed as suggested in the manual [74], with a standard deviation of split frequencies below 0.01, no obvious trend for the plot of the generation versus the log probability of the data, and the potential scale reduction factor (PSRF) close to 1.0 for all parameters.

### Genome sequencing and assembly

Genomic DNA was extracted as previously described [75]. The genomes of UM 1020 and UM 1400 were sequenced separately at different time periods. The sequencing and analysis of UM 1020 were reported previously as a genome announcement [6]. UM 1400 was sequenced with two libraries of 500 bp and 5 kb insert size, using Illumina HiSeq 2000 sequencer. The genome was assembled using Velvet version 1.2.07 [76] and the primary scaffolds from the Velvet assembly were further scaffolded with SSPACE Basic version 2.0 [77] and gap filled using GapFiller version 1.10 [78] utilizing paired-end information from both libraries. The genome completeness of both assemblies was accessed using CEGMA version 2.4 [18]. Whole genome alignments and comparison between the assembled scaffolds of both UM isolates were performed using NUCmer version 3.1 from MUMmer package version 3.23 [26] with default parameters. Genome assemblies of both UM isolates were then aligned and ordered using MAUVE version 2.3.1 [79]. Dot-plot was generated using mummerplot with the –color parameter and ordered set of UM 1020 genome sequence.

### Gene prediction and annotation

Gene prediction and functional annotation of both genome assemblies were carried out using the same methods to allow direct comparison. Prior to gene prediction, the repetitive elements, including interspersed repeats and low complexity DNA sequences were masked throughout the genomes using RepeatMasker version open-3.3.0 with the Repbase fungal library version rm-20,120,418. The *Ab initio* gene prediction was then performed on repeats-masked genomes using GeneMark-ES version 2.3 [80]. Predicted proteins were functionally annotated by local BLAST similarity searches against NCBI NR and SwissProt databases. Results from blast searches were analyzed for Gene Ontology (GO) and KEGG pathway using local Blast2GO tools [81]. Functional classification of the predicted proteins was performed using KOG [82]. Interpro analysis with Pfam database was used to annotate the predicted proteins based on protein domain families using InterProScan 5 [83]. The rRNA and tRNA of the genomes were predicted using RNAmmer version 1.2 [84] and tRNAscan-SE version 1.3.1 [85] respectively.

Predicted protein models were subjected to dbcan which is a web server and database for automated carbohydrate-active enzyme annotation [86]. Peptidases were identified by querying against MEROPS database release 9.9 using the batch blast service [87]. Secretome analysis was performed using the method as previously described [22], in which the predictions of cleavage sites and the signal peptide/non-signal peptide were carried out using SignalP version 4.1 [88]. Only secreted proteins without transmembrane (TM) domains and those with single TM present at the N-terminal 40 amino acids corresponding to secretion signals were selected. The presence of TM domains was identified using TMHMM version 2.0 [89]. Secondary metabolite backbone genes in both genomes were predicted using web-based software SMURF [90]. Pathogenicity-associated genes and stress response genes were identified by BLASTP search against local databases, which were downloaded and built from the pathogen-host interaction database (PHI-base) [91] and fungal stress response database (FSRD) [92] respectively, with criteria of BLASTP e-value threshold of less than or equal to 1e-5, percentage identity of more than 50 % and subject coverage of more than 70 %. Putative transposon elements were predicted using Transposon-PSI [93] which performed PSI-TBLASTN search of our genomes with a collection of (retro-)transposon ORF homology profiles to identify statistically significant alignments encoded by diverse families of transposable elements.

### Gene families

To identify orthologs of *D. eschscholtzii* within the class Sordariomycetes, protein sequences of all currently available Sordariomycetes genomes were downloaded from the databases. Proteins sequences (≥33 amino acids) from both UM isolates and the data-mined reference genomes were clustered using OrthoMCL software version 2.02 [94] which performs all-against-all BLASTP searches of all proteins, with reciprocal best blast hits from distinct genomes recognized as orthologs. OrthoMCL applied Markov Cluster algorithm [95] and was run with BLAST e-value cut-off of 1e-5 using an inflation parameter of 1.5.

### Phylogenomic analysis

For the phylogenomic tree construction, proteomes from Sordariomycetes fungi (used in gene family analysis) including our UM isolates were clustered using OrthoMCL software version 2.02 for within-class comparisons, with two Dothideomycetes fungi as outgroups. Individual multiple sequence alignments of 332 single-copy orthologs from the gene family analysis was generated using ClustalW version 2.0 [96]. The spurious sequences and poorly aligned regions were removed by using trimAL, and the multiple alignments were concatenated into a superalignment with 174,267 characters. Subsequently, ProtTest version 3.2 was run with AIC calculation on the alignment to select the best-fit substitution model [97]. A phylogenomic tree was constructed by using both RAxML version 7.7.9 [98] and MrBayes version 3.2.1 [73]. RAxML was run with model PROTGAMMAJTTF to search for the best-scoring Maximum Likelihood tree followed by 100 bootstrap replicates. Convergence was observed after 50 replicates using -I autoMRE option in RAxML. MrBayes was run using Jones amino acid model with gamma-distributed rate variation across sites and a proportion of invariable sites. The MCMC was run with a sampling frequency of 100 for 250,000 generations, and burn-in setting of 25 %.

## Additional files

Additional file 1: Table S1. Families of transposable elements identified in the genomes of *Daldinia eschscholtzii* UM 1400 and UM 1020​. **Table S2.** Information of the genome sequences used in phylogenomic and gene family analyses. **Table S3.** List of gene families and the distribution of the genes in each family among 11 Sordariomycetes fungi. **Table S4.** Gene families shared by both *Daldinia eschscholtzii* UM 1400 and UM 1020. **Table S5.** Number of CAZymes involved in plant polysaccharide degradation of 14 fungal genomes based on CAZyme database (used in Table 2). **Table S6.** List of predicted secondary metabolite backbone genes. **Table S7.** List of predicted genes encoding metallopeptidases (families M35 and M36) and subtilisin-like peptidases (subfamily S08A). **Table S8.** List of predicted genes encoding mitogen-activated protein kinase (MAPK) signaling components . **Table S9.** List of predicted genes encoding stress response proteins involved in adaptive responses to osmotic stress, pH stress, thermal stress, iron ion availability, nutrient stress, oxidative stress, and nitrosative stress for adaptation in harsh environment. (XLSX 1091 kb) Additional file 2: Figure S1. The distribution and classification of the putative PHI genes. (PDF 1241 kb)
