# Supplementary figures and images for: Genome analysis of Daldinia eschscholtzii strains UM 1400 and UM 1020, wood-decaying fungi isolated from human hosts
Source: BMC Genomics. 2015 Nov 18;16:966. doi: 10.1186/s12864-015-2200-2 (PMC4650942; doi:10.1186/s12864-015-2200-2)

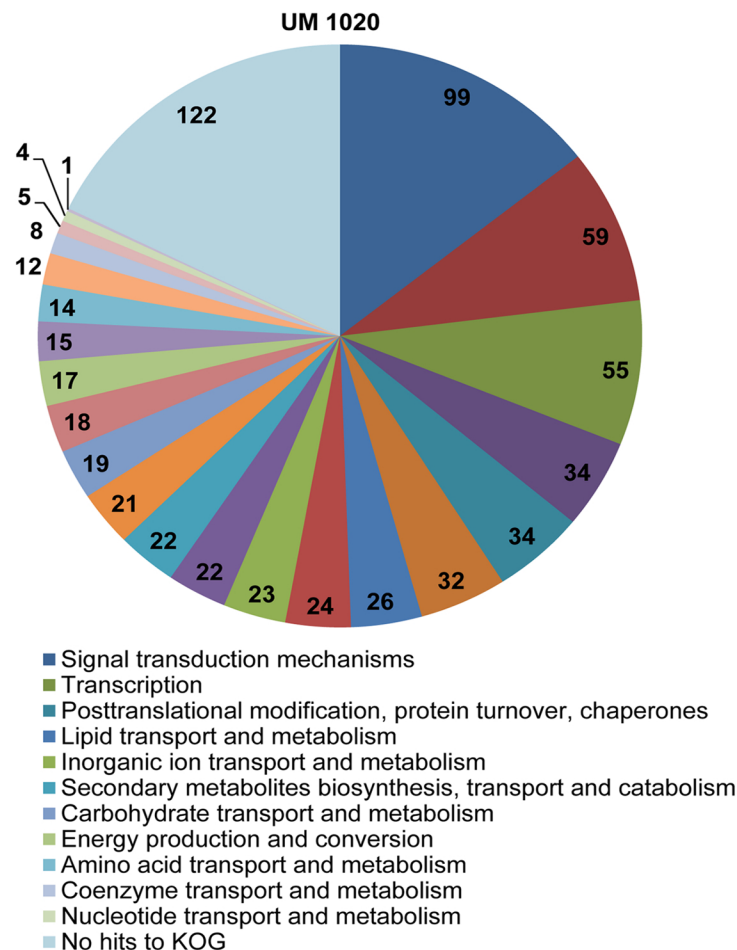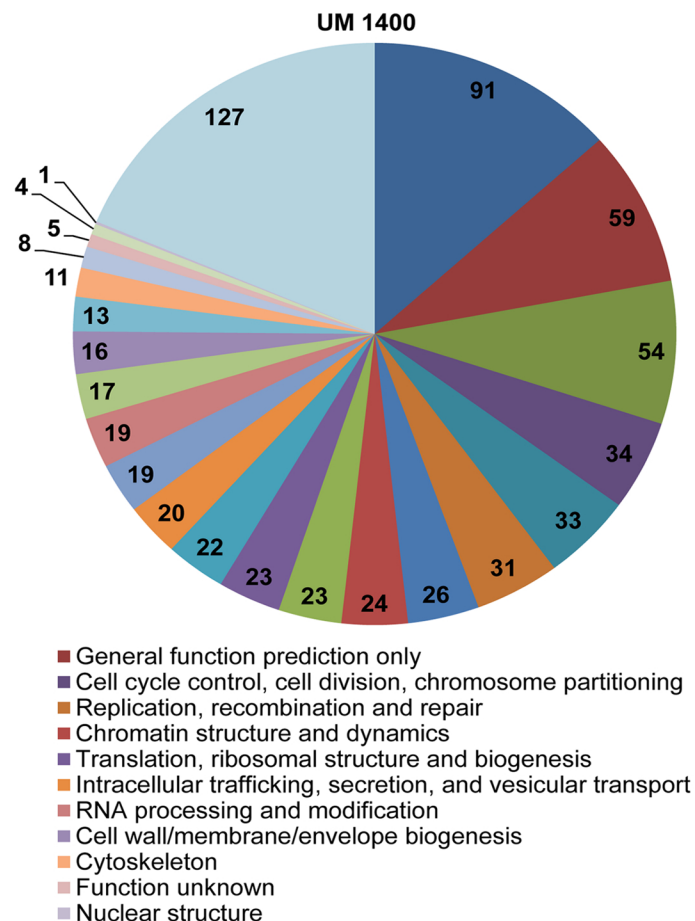

**Figure S1. The distribution and classification of the putative PHI genes.**

Supplement: Additional file 2: Figure S1. — The distribution and classification of the putative PHI genes. (PDF 1241 kb) [file 12864_2015_2200_MOESM2_ESM.pdf]
